# Correction: Bi-channel image registration and deep-learning segmentation (BIRDS) for efficient, versatile 3D mapping of mouse brain

**DOI:** 10.7554/eLife.74328

**Published:** 2021-10-05

**Authors:** Xuechun Wang, Weilin Zeng, Xiaodan Yang, Yongsheng Zhang, Chunyu Fang, Shaoqun Zeng, Yunyun Han, Peng Fei

Wang X, Zeng W, Yang X, Zhang Y, Fang C, Zeng S, Han Y, Fei P. 2021. Bi-channel image registration and deep-learning segmentation (BIRDS) for efficient, versatile 3D mapping of mouse brain. *eLife* **10**:e63455. doi: 10.7554/eLife.63455.

Published 18, January 2021

In our BIRDS software, a few parts in the registration module were contributed by Yongsheng Zhang and Prof. Shaoqun Zeng. When we were writing the eLife paper last year, we misunderstood that this work had been previously published along with their brain research work in their Wang et al 2020a paper (Science Bulletin 65:1203-1216) which we cited in the eLife paper. Due to this misunderstanding their contributions were only acknowledged in the published eLife paper. We are therefore formally correcting the eLife paper to add them both as co-authors. All the authors agree to their addition as co-authors in the paper.

The details of Yongsheng Zhang and Prof. Shaoqun Zeng’s contribution are explained below:

"Yongshen Zhang and Shaoqun Zeng first developed the efficient method to improve the registration stability by encoding global axis information using regional grayscale inversion. It allowed the extraction of more features from the raw image data and is indeed helpful in our BIRDS multi-channel registration framework; Yongsheng Zhang also developed the dynamic z down-sampling together with Weilin Zeng (exisiting author), to achieve better registration initialization. Furthermore, Yongsheng Zhang contributed to the development of interactive framework, which allows the operation of the local registration by drawing arrows on the merged coarse registered images instead of marking points, and notable improves the efficiency of semiautomatic registration. "

**New authors list:** Xuechun Wang, Weilin Zeng, Xiaodan Yang, **Yongsheng Zhang**, Chunyu Fang, **Shaoqun Zeng**, Yunyun Han, Peng Fei

**Original authors list:** Xuechun Wang, Weilin Zeng, Xiaodan Yang, Chunyu Fang, Yunyun Han, Peng Fei

Details for the omitted authors:

**Yongsheng Zhang**

^3^ Britton Chance Center for Biomedical Photonics, Wuhan National Laboratory for Optoelectronics, Huazhong University of Science and Technology, Wuhan, China.

**Contribution:** Methodology, Software

**For correspondence:** ys-zhang20@mails.tsinghua.edu.cn

**Contributed equally with:** Xuechun Wang, Weiling Zeng, Xiaodan Yang, Chunyu Fang (In the original published article ChunYu Fang was originally not a co-first author)

**Competing interests: **No competing interests exist

**Shaoqun Zeng**

^3^ Britton Chance Center for Biomedical Photonics, Wuhan National Laboratory for Optoelectronics, Huazhong University of Science and Technology, Wuhan, China.**Contribution:** Methodology

**Competing interests**: No competing interests exist

Revised author contributions:

Xuechun Wang: Data curation, Software, Validation, Visualization, Writing - original draft

Weilin Zeng: Methodology, Software, Validation

Xiaodan Yang: Resources, Visualization

Yong Sheng Zhang: Methodology, Software

Chunyu Fang: Resources

Shaoqun Zeng: Methodology

Yunyun Han: Resources, Visualization, Writing - review and editing

Peng Fei: Software, Funding acquisition, Visualization, Writing - review and editing

Original author contributions:

Xuechun Wang: Data curation, Software, Validation, Visualization, Methodology, Writing - original draft

Weilin Zeng: Software, Validation

Xiaodan Yang: Resources, Visualization

Chunyu Fang, Resources, Visualization

Yunyun Han, Resources, Visualization, Writing - review and editing

Peng Fei, Software, Funding acquisition, Visualization, Writing - review and editing

Revised funding statement:

**Funding**

National Natural Science Foundation of China (21874052)

Peng Fei

National Natural Science Foundation of China (31871089)

Yunyun Han

Innovation Fund of WNLO

Peng Fei

Junior Thousand Talents Program of China

Yunyun Han

Peng Fei

The FRFCU (HUST:2172019kfyXKJC077)

Yunyun Han

National Key R&D program of China (2017YFA0700501)

Peng Fei

973 Project (2015CB755603)

Shaoqun Zeng

Yongsheng Zhang

Director Fund of WNLO

Shaoqun Zeng

Yongsheng Zhang

Original funding statement:

Funding

National Natural Science Foundation of China (21874052)

Peng Fei

National Natural Science Foundation of China (31871089)

Yunyun Han

Innovation Fund of WNLO

Peng Fei

Junior Thousand Talents Program of China

Yunyun Han

Peng Fei

The FRFCU (HUST:2172019kfyXKJC077)

Yunyun Han

National Key R&D program of China (2017YFA0700501)

Peng Fei

The funders had no role in study design, data collection and interpretation, or the decision to submit the work for publication.

**Revised Acknowledgements:**

We thank Haohong Li, Luoying Zhang, Man Jiang, Bo Xiong for discussions and comments on the work and Hao Zhang for the help on the code implementation. This work was supported by the National Key R and D program of China (2017YFA0700501 PF), the National Natural Science Foundation of China (21874052 for PF, 31871089 for YH), the Innovation Fund of WNLO (PF) and the Junior Thousand Talents Program of China (PF and YH), the FRFCU (HUST:2172019kfyXKJC077 YH).

**Original Acknowledgements:**

We thank Yongsheng Zhang, Shaoqun Zeng, Haohong Li, Luoying Zhang, Man Jiang, Bo Xiong for discussions and comments on the work. Hao Zhang for the help on the code implementation. This work was supported by the National Key R and D program of China (2017YFA0700501 PF), the National Natural Science Foundation of China (21874052 for PF,31871089 for YH), the Innovation Fund of WNLO (PF) and the Junior Thousand Talents Program of China (PF and YH), the FRFCU (HUST:2172019kfyXKJC077 YH).

The article has been corrected accordingly.

